# *Ishige okamurae* Extract and Its Constituent Ishophloroglucin A Attenuated In Vitro and In Vivo High Glucose-Induced Angiogenesis

**DOI:** 10.3390/ijms20225542

**Published:** 2019-11-06

**Authors:** K.H.N. Fernando, Hye-Won Yang, Yunfei Jiang, You-Jin Jeon, BoMi Ryu

**Affiliations:** 1 Department of Marine Life Science, School of Marine Biomedical Sciences, Jeju National University, Jeju 63243, Koreakoty221@naver.com (H.-W.Y.); jiangyunfei0310@gmail.com (Y.J.); 2 Marine Science Institute, Jeju National University, Jeju 63333, Korea

**Keywords:** *Ishige okamurae*, Ishophloroglucin A, diabetes, angiogenesis

## Abstract

Diabetes is associated with vascular complications, such as impaired wound healing and accelerated vascular growth. The different clinical manifestations, such as retinopathy and nephropathy, reveal the severity of enhanced vascular growth known as angiogenesis. This study was performed to evaluate the effects of an extract of *Ishige okamurae* (IO) and its constituent, Ishophloroglucin A (IPA) on high glucose-induced angiogenesis. A transgenic zebrafish (*flk*:EGFP) embryo model was used to evaluate vessel growth. The 3-(4,5-Dimethylthiazol-2-yl)-2,5-diphenyltetrazolium bromide (MTT), gap closure, transwell, and Matrigel^®^ assays were used to analyze the proliferation, migration, and capillary formation of EA.hy926 cells. Moreover, protein expression were determined using western blotting. IO extract and IPA suppressed vessel formation in the transgenic zebrafish (*flk*:EGFP) embryo. IPA attenuated cell proliferation, cell migration, and capillary-like structure formation in high glucose-treated human vascular endothelial cells. Further, IPA down regulated the expression of high glucose-induced vascular endothelial growth factor receptor 2 (VEGFR-2) and downstream signaling molecule cascade. Overall, the IO extract and IPA exhibited anti-angiogenic effects against high glucose-induced angiogenesis, suggesting their potential for use as therapeutic agents in diabetes-related angiogenesis.

## 1. Introduction

Diabetes is associated with secondary metabolic complications, such as insulin resistance and hyperinsulinemia, leading to abnormal angiogenesis [1]. Development of new microvessels from the existing vessels is known as angiogenesis [2]. Diabetes is characterized by inadequate angiogenesis in some organs and excessive angiogenesis in some others [3]. Apart from its role in several pathological conditions, angiogenesis also plays a crucial role in normal growth and development [4]. Excessive angiogenesis causes the degradation of vascular endothelial cells from the extracellular matrix, enhancement of cell proliferation, migration, and formation of extravascular networks [5]. Excessive angiogenesis is observed in diabetic retinopathy and nephropathy, resulting in the loss of vision and renal failure, respectively [6].

Marine algae are considered a prolific source of important bioactive compounds that aid in maintaining normal health and mitigating disease risks [7]. Among the marine algae, brown algae and its constituent phlorotannins are widely studied globally for various biological effects by several research groups [8]. *Ishige okamurae* (IO) is an edible brown alga found abundantly in the coastal areas of Jeju Island. It has been reported that IO exerts several biological activities, such as anti-α-glucosidase, free-radical scavenging, cytoprotective, anti-obesity, and anti-inflammatory activities [9,10,11]. Diphlorethohydroxycarmalol (DPHC), also a kind of phlorotannin isolated from IO extract has been studied in our previous work [11], and it exhibited anti-angiogenic effects against high glucose-induced angiogenesis. Ishophloroglucin A (IPA) is a novel phlorotannin isolated from IO extract, which has been studied for standardizing the anti-α-glucosidase activity of IO [12]. However, the effects of IO extract and IPA in the context of diabetic-related pathologies have not been examined. Therefore, in the present study, IO extract and IPA were studied for their anti-angiogenic effects on high glucose-induced vascular growth.

The zebrafish model is widely used in studies on angiogenesis due to its characteristics. Transgenic zebrafish lines are more suitable for imaging of the vessels with fluorescent labeling and the alterations can be clearly visualized [13]. In this study, we used transgenic zebrafish Tg (*flk:EGFP*), which has fluorescently-labeled complete vasculature and is widely used for screening anti-angiogenic compounds [14]. The vascular endothelium is a biologically-important layer present in the blood vessels, and its dysfunction results in various vascular pathologies [6]. The EA.hy926 cell line is frequently used in different angiogenesis studies. It is established by the fusion of primary human umbilical vein endothelial cells (HUVEC) and human lung carcinoma cell line A549 [15]. These cells are more appropriate than primary vascular cells because they are mortal and do not possess variations associated with the donor [16]. The cell line EA.hy926 has been experimented for vascular endothelial cell characteristics and is known to possess characters of macro and micro vessels [16]. In this study, the anti-angiogenic effects of IPA were evaluated in EA.hy926 cells. We also investigated cell proliferation, cell migration, and capillary-like structure formation in high glucose-treated EA.hy926 cells.

In diabetes-induced angiogenesis, vascular endothelial growth factor receptor 2 (VEGFR-2) is activated, and the downstream signaling events associated with its activation play a significant role in angiogenesis [17]. Therefore, the expression of VEGFR-2 and its downstream signaling molecules were evaluated to elucidate the mechanisms by which IPA affects high glucose-induced angiogenesis.

## 2. Results

### 2.1. Effects of IO Extract on High Glucose-Treated Zebrafish Embryo

**Figure 1:**
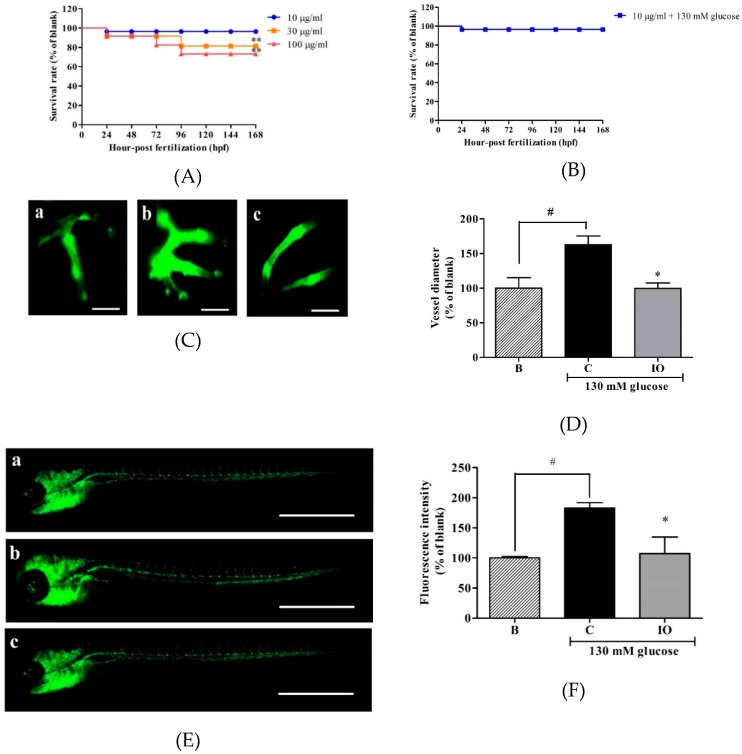
Effects of *Ishige okamurae* (IO) extract on zebrafish embryo. (**A**) Effects of IO extract on the survival rate of transgenic zebrafish (*flk*:EGFP) embryos. The embryos were treated with 10, 30, and 100 µg/mL of IO extract for 24, 48, 72, 96, 120, 144, and 168 h post fertilization (hpf). The treatment effects were normalized to blank (0 µg/mL IO extract). (**B**) The survival rate of transgenic zebrafish (flk:EGFP) embryos treated with 10 µg/mL IO extract and 130 mM glucose together. (**C**) Fluorescence microscopic images of the retinal vessels of transgenic zebrafish (*flk*:EGFP) embryos treated with IO extract. (**D**) The diameter of the hyaloid retinal vessel treated with IO extract. (**E**) Images of the whole body vessel formation in transgenic zebrafish (*flk*:EGFP) embryos treated with IO extract as obtained by fluorescence microscopy. (**F**) Quantified fluorescence intensity of the whole body treated with IO extract (a: 0 mM glucose + 0 µg/mL IO, b: 130 mM glucose + 0 µg/mL IO, and c: 130 mM glucose + 10 µg/mL IO). The effects of 130 mM glucose on vessel formation were compared with B (blank (0 mM glucose + 0 µg/mL IO extract)). The effects of Ishophloroglucin A (IPA) on high glucose-induced vessel formation were normalized to C (control (130 mM glucose + 0 µg/mL IO extract)). Scale bar (C) 20 µm, (E) 1000 µm. * *p* ˂ 0.05, ^#^ *p* ˂ 0.05.

The toxicity of IO extract in transgenic zebrafish (*flk*:EGFP) embryo was investigated using different concentrations of IO extract (10, 30, and 100 µg/mL). As shown in Figure 1A, 10 µg/mL IO extract showed no significant toxicity in transgenic zebrafish (*flk*:EGFP) embryo. Furthermore, there was no toxicity following treatment with 10 µg/mL and 130 mM glucose together (Figure 1B). Hence, this concentration was used in further experiments. 

Transgenic zebrafish (*flk*:EGFP) embryos were treated with 130 mM glucose [11] to induce angiogenesis in the whole body, including hyaloid-retinal vessels (Figure 1). Treatment with glucose (130 mM) yielded 162.7% of retinal vessel compared with that of the blank (no glucose). Treatment with 10 µg/mL IO extract significantly suppressed the retinal vessel diameter (99.5% similar to the blank) (Figure 1C,D). Fluorescence intensity was measured for the quantitative analysis of vascular growth in the whole body. Treatment with glucose (130 mM) yielded 182.8% fluorescence intensity compared with that of the blank (Figure 1E,F). Treatment with 10 µg/mL IO extract suppressed the high glucose-induced vascular growth in the whole body (107.1% intensity compared with that of the blank).

### 2.2. Effects of IPA on High Glucose-Treated Zebrafish Embryo

**Figure 2:**
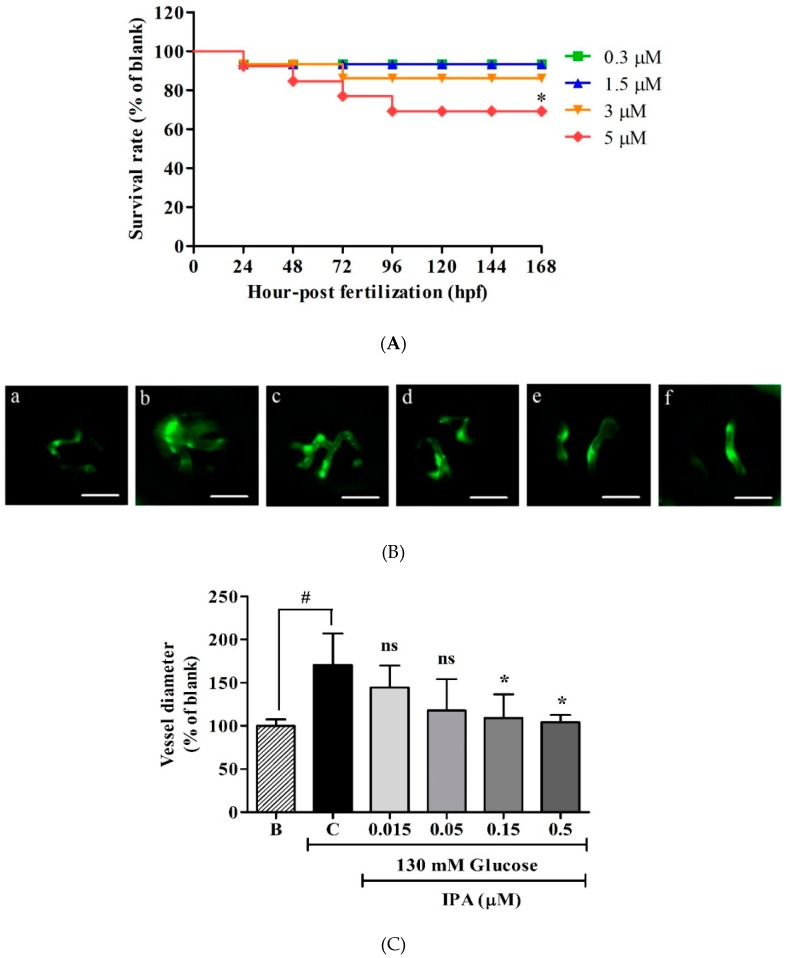

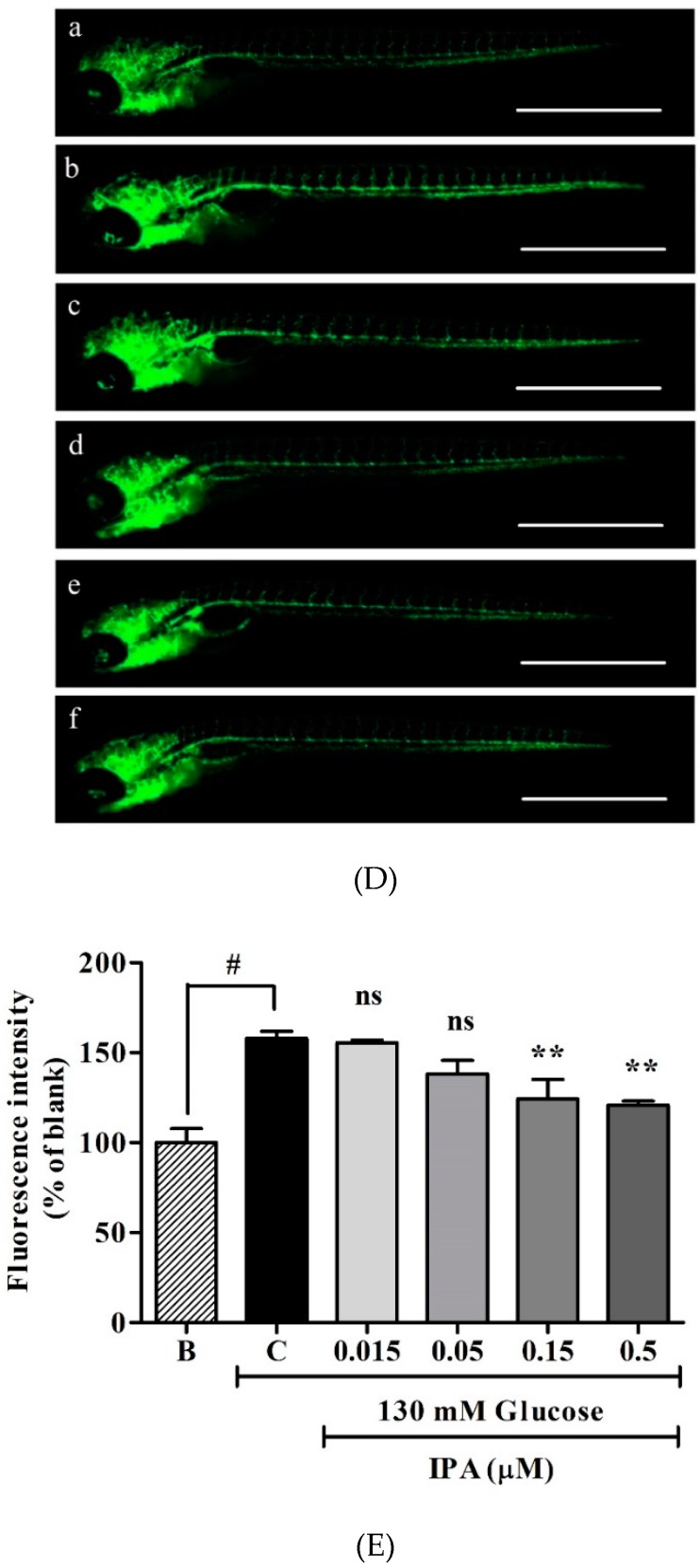
Effects of IPA on transgenic zebrafish (*flk*:EGFP) embryos. (**A**) Effects of IPA on the survival rate of transgenic zebrafish (*flk*:EGFP) embryos. The embryos were treated with 0.3, 1.5, 3, and 5 µM IPA for 24, 48, 72, 96, 120, 144, and 168 hpf. The treatment effects were normalized to blank (0 µM IPA). (**B**) Fluorescence microscopic images of the retinal vessels of transgenic zebrafish (*flk*:EGFP) embryos treated with IPA. (**C**) The diameter of hyaloid retinal vessels treated with IPA. (**D**) The images of whole body vessel formation in transgenic zebrafish (*flk*:EGFP) embryos treated with IPA as obtained by fluorescence microscopy. (**E**) Quantified fluorescence intensity of the whole body treated with IO extract (a: 0 mM glucose + 0 µM IPA, b: 130 mM glucose + 0 µM IPA, c: 130 mM glucose + 0.05 µM IPA, d: 130 mM glucose + 0.15 µM IPA, e: 130 mM glucose + 0.5 µM IPA, and f: 130 mM glucose + 1.5 µM IPA). The effects of 130 mM glucose on vessel formation were compared to B (blank (0 mM glucose + 0 µM IPA)). The effects of IPA on high glucose-induced vessel formation were normalized to C (control (130 mM glucose + 0 µM IPA)). Scale bar (B) 20 µm, (D) 1000 µm. ns; not significant, * *p* ˂ 0.05, ** *p* ˂ 0.01, ^#^ *p* ˂ 0.05.

Initially, the toxicity of IPA on transgenic zebrafish (*flk*:EGFP) embryo was investigated with different concentrations of IPA (0.3, 1.5, 3, and 5 µM). The results showed (Figure 2A) that IPA at concentrations of up to 3 µM had no significant toxic effects. Hence, we selected IPA concentrations of 0.015, 0.05, 0.15, and 0.5 µM to evaluate the anti-angiogenic effects in transgenic zebrafish (*flk*:EGFP) embryo.

Glucose treatment yielded 170.4% retinal vessel. When treated with IPA at concentrations of 0.015, 0.05, 0.15, and 0.5 µM, the retinal vessel diameters were decreased to 144.49%, 117.87%, 109.14%, and 104.36%, respectively, compared with that of the blank (Figure 2B,C). The fluorescence intensity of glucose treatment was 157.8%. Following treatment with IPA at concentrations of 0.15 and 0.5 µM, the fluorescence intensity significantly decreased to 124.43% and 120.9%, validating the anti-angiogenesis effect of 10 µg/mL IO extract with 0.0907 µM IPA (Figure 2D,E). After observing vascular growth in the hyaloid-retina and the whole body, it could be inferred that treatment with IPA may lead to anti-angiogenic effects against high glucose-induced angiogenesis.

### 2.3. Effects of IPA on High Glucose-Induced Cell Proliferation, Migration, and Capillary-Like Structure Formation 

**Figure 3:**
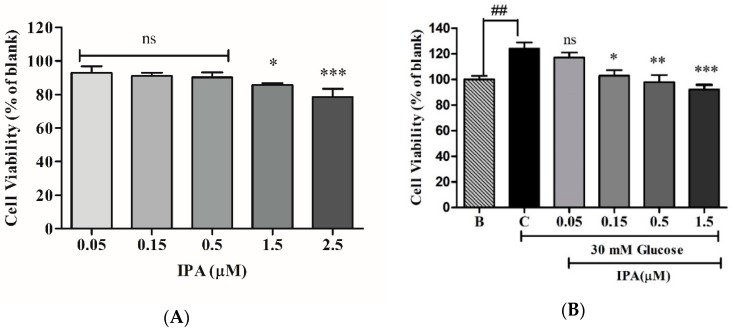
IPA inhibits the proliferation of EA.hy926 cells. (**A**) Cytotoxicity of IPA in EA.hy926 cells. The cells were treated with different IPA concentrations (0, 0.05, 0.15, 0.5, 1.5, and 2.5 µM) for 24 h, and the cell viability was determined by 3-(4,5-Dimethylthiazol-2-yl)-2,5-diphenyltetrazolium bromide (MTT) assay. The results were normalized to blank (0 µM IPA). (**B**) IPA inhibits the proliferation of high glucose-induced EA.hy926 cells. The cells were treated with different concentrations of IPA (0.05, 0.15, 0.5, and 1.5 µM), along with 30 mM glucose. MTT assay was performed to determine cell viability. The anti-proliferative effects of IPA in high glucose-induced cells were normalized to C (control (30 mM glucose + 0 µM IPA)), and the effects of 30 mM glucose were compared to B (blank (0 mM glucose + 0 µM IPA)). ns; not significant, * *p* ˂ 0.05, ** *p* ˂ 0.01, *** *p* ˂ 0.001, ^##^ *p* ˂ 0.01.

Prior to assessing the anti-angiogenic effects of IPA, the 3-(4,5-Dimethylthiazol-2-yl)-2,5-diphenyltetrazolium bromide (MTT) assay was performed to evaluate its cytotoxicity in EA.hy926 cells. The cell viability was 92.94%, 91.31%, 90.24%, 86.78%, and 78.48% when treated with IPA at concentrations of 0.05, 0.15, 0.5, 1.5, and 2.5 µM, respectively (Figure 3A). The non-toxic IPA concentrations of 0.05, 0.15, 0.5, and 1.5 µM were used in later experiments, as >80% cell viability was selected for use in the cellular experiments [18]. The anti-angiogenesis effect of IPA was evaluated with regard to cell proliferation, cell migration, and capillary formation. The cell viability was used as an indicator of cell proliferation, while in our previous study [11], we used Muse™ Cell Analyzer to confirm the significant cell proliferation at 30 mM glucose treatment. As shown in Figure 3B, significant cell proliferation (124.93%) was observed after treatment with 30 mM glucose. Once the cells were treated together with 30 mM glucose and ascending concentrations of IPA, cell proliferation was decreased significantly in a concentration-dependent manner. The results were 117.12%, 102.95%, 97.80%, and 92.21% when treated with IPA at concentrations of 0.05, 0.15, 0.5, and 1.5 µM, respectively. These results suggest that IPA exerts anti-angiogenic effects by inhibiting high glucose-induced vascular cell proliferation. 

**Figure 4:**
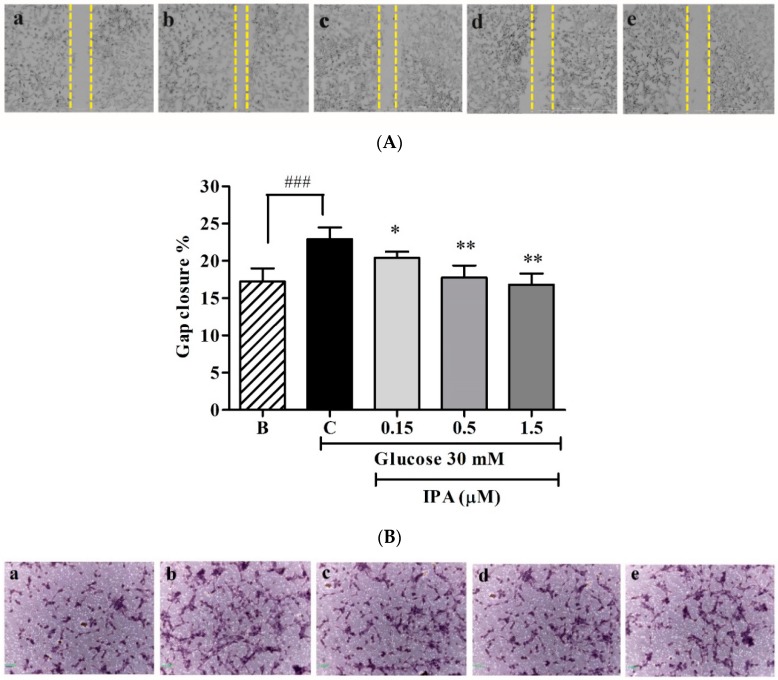

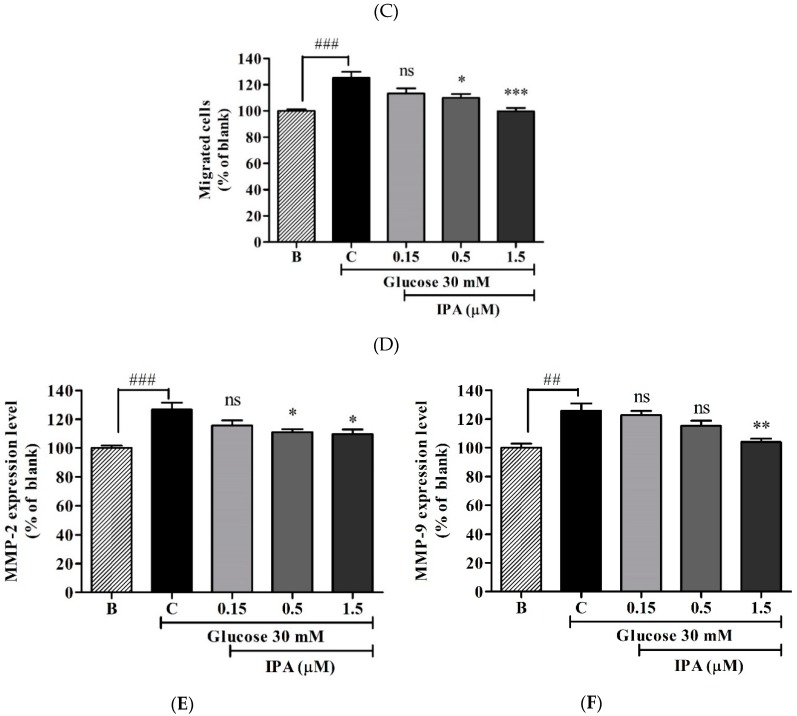
(**A**) IPA inhibits high glucose-induced cell migration. The cells were treated with different concentrations of IPA (0.15, 0.5, and 1.5 µM), along with 30 mM glucose. The cell monolayer was scraped at the middle of the well, and the initial gap length (0 h) and the final gap length (12 h) was measured. (**B**) The gap closure was quantified by percentage (%). (**C**) IPA inhibits cell migration through transwell filter chambers. The migrated cells were fixed, stained, and counted. (**D**) Quantification of migrated cells through transwell filter chambers. Matrix Metalloproteinase (MMP) (MMP-2 and -9) expression levels were evaluated using commercial Enzyme-Linked Immunosorbent Assay (ELISA) kits. (**E**) Quantification of MMP-2 expression. (**F**) Quantification of MMP-9 expression. The effects of 30 mM glucose on cell migration/MMP expression levels were compared to B (blank (0 mM glucose + 0 µM IPA)), and the effects of IPA on high glucose-induced cell migration/MMP expression level were normalized to C (control (30 mM glucose + 0 µM IPA)). Scale bar (A) and (C) 1000 µm. ns; not significant, * *p* ˂ 0.05, ** *p* ˂ 0.01, *** *p* ˂ 0.001, ^##^ *p* ˂ 0.01, ^###^ *p* ˂ 0.001.

The scratch-wound cell migration and transwell migration assays were used to determine the effects of IPA on high glucose-induced cell migration. In the scratch-wound cell migration assay, the cell migration ability was compared by calculating the gap closure percentage (Figure 4A,B). The higher gap closure percentage indicated higher cell migration ability and vice versa. The highest cell migration recorded was 22.91% after treatment with 30 mM glucose. It was significantly decreased, by 20.42%, 17.76%, and 16.8%, following treatment with IPA at concentrations of 0.15, 0.5, and 1.5 µM, respectively.

A similar result was obtained in the transwell migration assay. The percentage of cell migration through the transwell was higher when the cells were treated with 30 mM glucose than that under normal glucose condition. With IPA treatment, high glucose-induced cell migration was inhibited significantly in a dose-dependent manner (Figure 4C,D). The migrated cell percentage was 113.33%, 110.54%, and 99.66% when treated with IPA at concentrations of 0.15, 0.5, and 1.5 µM, respectively. These observations indicated that IPA effectively suppressed high glucose-induced cell migration.

Cell migration was further validated by quantification of Matrix Metalloproteinases (MMPs) using the Enzyme-Linked Immunosorbent Assay (ELISA). MMP-2 and -9 were significantly high in the high glucose treatment, and IPA treatment reduced both MMP-2 and -9 in the cell media (Figure 4E,F). MMP-2 percentage was 115.12%, 111.25%, and 109.42% following treatment with IPA at concentrations of 0.15, 0.5, and 1.5 µM, respectively, whereas the MMP-9 percentage was 122.58%, 115.45%, and 104.36% following treatment with IPA at concentrations of 0.15, 0.5, and 1.5 µM, respectively.

**Figure 5:**
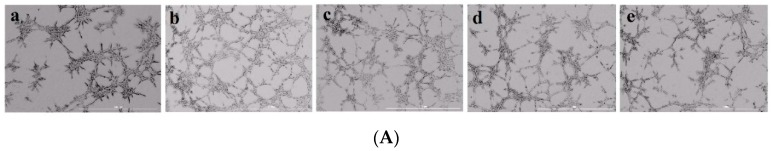

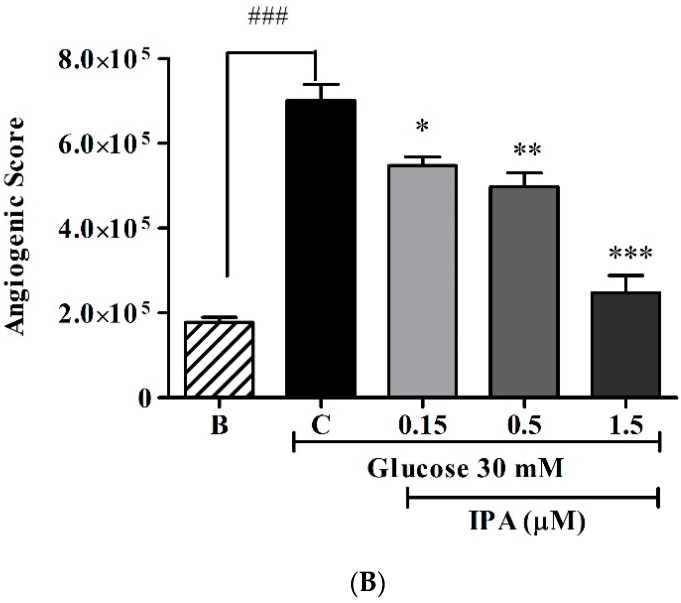
(**A**) IPA suppresses high glucose-induced capillary formation in Matrigel^®^. The cells were seeded on Matrigel^®^ with different concentrations of IPA (0.15, 0.5, and 1.5 µM), along with 30 mM glucose including B (blank (0 mM glucose + 0 µM IPA)) and C (control (30 mM glucose + 0 µM IPA)). After 6 h incubation, the cells were photographed and the angiogenic score was determined. (**B**) Quantification of capillary formation. The effects of 30 mM glucose on capillary formation were compared to B (blank (0 mM glucose + 0 µM IPA)). The effects of IPA on high glucose-induced capillary formation were normalized to C (control (30 mM glucose + 0 µM IPA)). Scale bar (A) 1000 µm. * *p* ˂ 0.05, ** *p* ˂ 0.01, *** *p* ˂ 0.001, ^###^ *p* ˂ 0.001.

Vascular endothelial cells cultured in Matrigel^®^ matrix can differentiate into capillary-like structures [19]. This characteristic feature was used to evaluate the IPA effects on high glucose-induced capillary-like structure formation (Figure 5A,B). The angiogenic score was determined for quantitative evaluation of capillary formation. An increased angiogenic score is an indicator of higher capillary formation. According to the results, a higher angiogenic score was reported as 7.01 × 10^5^ in the cells treated with 30 mM glucose. After IPA treatment, the angiogenic score was significantly decreased. The angiogenic score was 5.47 × 10^5^, 4.97 × 10^5^, and 2.47 × 10^5^ when treated with IPA at concentrations of 0.15, 0.5, and 1.5 µM, respectively. These data suggest that IPA exerts anti-angiogenic effects by suppressing capillary formation. 

### 2.4. Effects of IPA on VEGFR-2 and the Downstream Signaling Cascade

**Figure 6:**
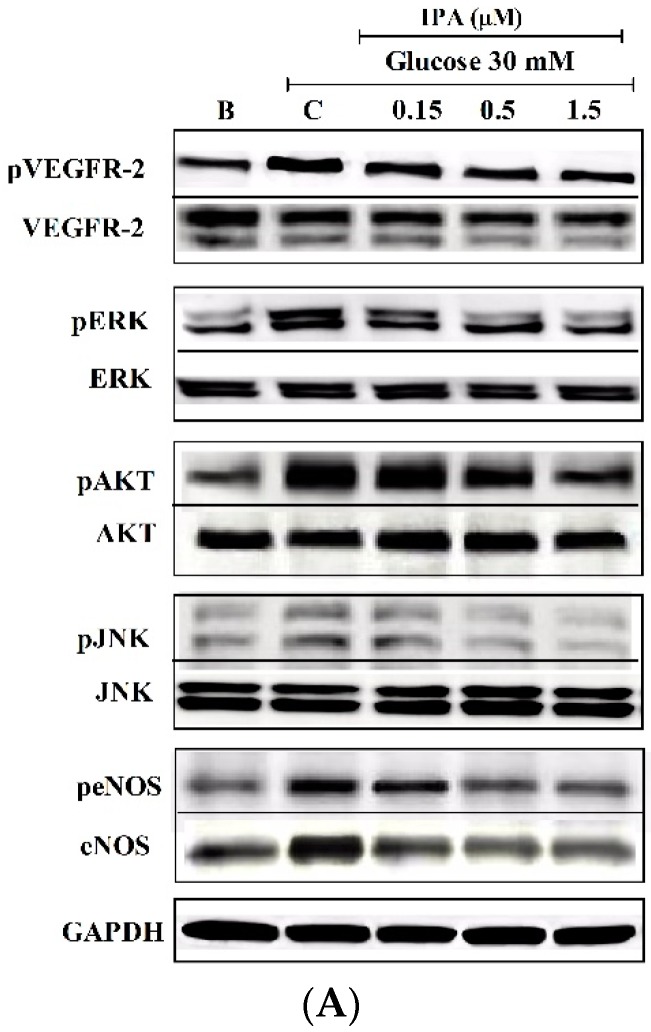

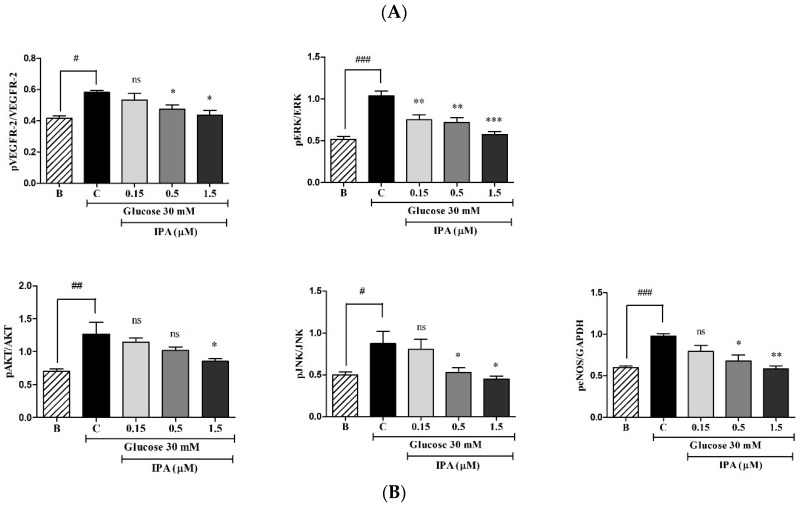
(**A**) IPA inhibits vascular endothelial growth factor receptor 2 (VEGFR-2) and its downstream signaling molecules. IPA attenuating high glucose-induced phosphorylation of VEGFR-2 and downstream signaling molecules extracellular signal-regulated kinase (ERK), protein kinase B (AKT), c-Jun N-terminal kinase (JNK), and endothelial nitric oxide synthase (eNOS) were detected using western blotting. (**B**) Quantitative evaluation of the protein expression. The effects of 30 mM glucose on the expression of each protein were compared to B (blank (0 mM glucose + 0 µM IPA)). The effects of IPA on high glucose-induced protein expression were normalized to C (control (30 mM glucose + 0 µM IPA)). ns; not significant, * *p* ˂ 0.05, ** *p* ˂ 0.01, *** *p* ˂ 0.001, ^#^ *p* ˂ 0.05, ^##^ *p* ˂ 0.01, ^###^ *p* ˂ 0.001.

The expression of pVEGFR2 and its downstream signaling molecules were detected by western blotting (Figure 6). pVEGFR-2 expression was significantly increased in the high glucose-treated EA.hy926 cells compared with that of the blank. As shown in Figure 6B, high glucose-induced pVEGFR-2 expression was decreased significantly in the cells treated with IPA. In addition, high glucose treatment showed higher protein expression in the downstream signaling molecules extracellular signal-regulated kinase (ERK), protein kinase B (AKT), c-Jun N-terminal kinase (JNK), and endothelial nitric oxide synthase (eNOS). With IPA treatment, these parameters were significantly down regulated. 

## 3. Discussion

Studies have demonstrated that seaweeds are rich in bioactive components with medicinal values [20]. IO has long been used as an edible seaweed in Korea. Previous studies have demonstrated the potential of the ethanolic extract of IO to treat chronic inflammation [21]. A recent study has shown that the ethanolic extract of IO possesses anti-diabetic activities by inhibiting α-glucosidase [12]. To the best of our knowledge, there has been no study demonstrating the anti-angiogenic effects of the ethanolic extract of IO. Here, for the first time, we demonstrated the anti-angiogenic effects of the ethanolic extract of IO on high glucose-induced-angiogenesis.

IPA is a phlorotannin isolated from the IO extract, and it is known for its α-glucosidase inhibitory activity and constitutes 1.81% ± 0.362 of IO [12] (Supplementary Figure S1). Treatment with 10 µg/mL of IO extract showed anti-angiogenic effects against high glucose-induced vascular growth. Based on this observation, we hypothesized that IPA from IO extract could be a key molecule involved in the anti-angiogenic effects.

IPA contains hydroxyl groups bonded with its benzene structure (Supplementary Figure S2). The comparatively higher number of hydroxyl groups may be advantageous to its biological activities. It has been reported that phlorotannins, which contain > 10 hydroxyl groups, show relatively high anti-oxidant activities [22]. Analysis of our data revealed that IPA exerted anti-angiogenic effects in the concentration range of 0.05−0.15 µM. This represents approximately 0.1−0.3 µg/mL of IPA (molecular weight of IPA, 1984 g/mol). Therefore, the anti-angiogenic effects of IO extract could be attributed to the IPA present in the IO extract. Further studies were carried out with IPA in vascular endothelial cells EA.hy926 to evaluate the cellular mechanisms against high glucose-induced angiogenesis. 

Angiogenesis is a step-by-step process involving cell proliferation, migration, and capillary formation [23]. In angiogenesis, cell migration is an essential event where the cells move towards a controlled direction before capillary morphogenesis [24] and, in high glucose treatment, cell migration is also increased [25]. IPA significantly reduced the high glucose-induced cell migration in the scratch wound migration and transwell migration assays. This was further validated via inhibition of MMP-2 and -9. The MMPs are a kind of proteases that are critically important in degrading the extracellular matrix (ECM) to facilitate endothelial cell migration in the angiogenesis process [26]. Among various MMPs, MMP-2 and -9 more efficiently degrade basement membrane components [27]. Furthermore, capillary formation was evaluated because, in the process of developing drugs targeting angiogenesis, the 3D capillary formation is an important aspect [28]. Overall, our data showed that IPA was efficacious in inhibiting high glucose-induced endothelial cell proliferation, migration, and capillary formation.

In mammals, there are three different VEGF receptors, namely, VEGFR-1, VEGFR-2, and VEGFR-3. VEGFR-1 is important for hematopoietic cell development, and VEGFR-3 is crucial for lymphatic endothelial cell development. VEGFR-2 is the principal receptor involved in endothelial cell development and has attracted considerable attention in the anti-angiogenic therapeutic intervention [17]. Bevacizumab is an example of a drug that targets VEGFR inhibition, although it causes several adverse effects, such as hypertension, fatigue, rash, and myalgia, due to lack of target specificity [29]. Therefore, today, interventions by anti-angiogenic drugs obtained from natural compounds are preferred because of the low adverse effects profile. Qi et al. [30] reported the anti-angiogenic effects of bromophenol bis(2,3-dibromo-4,5-dihydroxybenzyl) ether from a marine source in vascular endothelial cells by suppressing the VEGFR signaling pathway. Further, Lu and Basu [31] studied chebulagic acid, a polyphenol of myrobalan fruits that suppressed the VEGFR-2 phosphorylation and inhibited the angiogenesis in vascular endothelial cells.

According to previous supporting evidence [32], downstream signaling mediators of VEGFR-2, including ERK, AKT, JNK, and eNOS, are involved in the regulation of endothelial cell proliferation and survival. ERK and JNK are actively involved in endothelial cell proliferation [33], whereas AKT plays an important role in endothelial cell survival [34]. eNOS is involved in the production of large amounts of nitric oxide (NO) in the endothelial cells and plays a critical role in all the processes of angiogenesis, including matrix breakup, endothelial cell migration, proliferation, network structure organization, and lumen formation [35]. Our results demonstrated that IPA, isolated from a marine alga, exerts its anti-angiogenic effects by interfering with the VEGFR-2 signaling pathway.

## 4. Materials and Methods 

### 4.1. Materials

Human vascular endothelial cell line EA.hy926 was obtained from the American Type Culture Collection (ATCC, Rockville, MD, USA). Dulbecco’s modified Eagle medium (DMEM) and penicillin–streptomycin mixture were purchased from Gibco Life Technologies (Grand Island, NY, USA). Fetal bovine serum (FBS) was purchased from Merck (Sacramento, CA, USA). 3-(4, 5-Dimethylthiazol-2-yl)-2,5-diphenyltetrazolium bromide (MTT) was obtained from VWR Life Science (Lutterworth, UK). Dimethyl sulfoxide (DMSO) was purchased from Amresco Life Sciences (Solon, OH, USA). Transwell plates (8 µM) were purchased from SPL (SPL Life Sciences, Pocheon, Korea). Human MMP-2 and -9 ELISA kits were purchased from Sigma-Aldrich (St. Louis, MO, USA). Matrigel^®^ was obtained from BD Biosciences (Bedford, MA, USA). Both primary and secondary antibodies used in western blotting were purchased from Santa Cruz Biotechnology (Santa Cruz, CA, USA). All other chemicals were commercially available and analytical grade.

### 4.2. Preparation of IO Extract and IPA

*Ishige okamurae* was collected in April 2016 in Seongsan, Jeju Island, South Korea. IO extract was prepared and IPA isolated using a previously described method [12]. Briefly, 50% ethanolic extract of IO was fractionated using centrifugal partition chromatography (CPC 240, Tokyo, Japan) and further purified using semipreparative HPLC column (YMC-Pack ODS-A; 10 mm × 250 mm, 5µm) to obtain IPA. The identity of IPA (99% of purity) was verified using MS fragmentation of *m*/*z* 1986.26 using ultrahigh resolution Q-TOF LC-MS/MS coupled with an electrospray ionization (ESI) resource (maXis-HD; Bruker Daltonics, Breman, Germany) at the Korea Basic Science Institute (KBSI) in Ochang, South Korea. According to a previously validated method [12], the IO extract used in this study had 1.81% ± 0.362 IPA.

### 4.3. Treatment of Zebrafish Transgenic (flk:EGFP) Embryos with IO Extract and IPA

Before assessing the anti-angiogenesis effect, the survival rate following treatment with IO extract and IPA was determined in zebrafish transgenic (*flk*:EGFP) embryos. Five embryos were placed in each well of 24-well plates and maintained in embryonic water containing different concentrations of IO extract (10, 30, and 100 µg/mL) or IPA (0.3, 1.5, 3 and 5 µM), and the survival rate was assessed for 168 h post fertilization (hpf). The survival rate of zebrafish transgenic (*flk*:EGFP) embryos after treatment with 10 µg/mL IO extract and 130 mM glucose was determined. 

### 4.4. Zebrafish Transgenic (flk:EGFP) Embryos and Angiogenesis Assay

Zebrafish embryos with high glucose-induced angiogenesis was developed as described previously [36] by maintaining zebrafish transgenic (*flk*:EGFP) embryos (3 days post fertilization (dpf)) in embryonic water containing 130 mM glucose for 3 days. High glucose-treated embryos were treated with 10 µg/mL of IO extract or different concentrations of IPA (0.05, 0.15, 0.5, and 1.5 µM), and vessel growth was examined in hyaloid retinal vessels and in the whole body. After 3 h of treatment, images were captured using a fluorescence microscope (LIONHEART FX automated live cell imager). Vessel formation in retinal vessels was evaluated by measuring the retinal vessel diameter of the images (10× magnification) at five different places using Gen5 3.04 software, and then it was averaged. Vessel formation in the whole body was assessed by measuring the fluorescence intensities of the images (4× magnification) using Image J software, followed by calculation of the corrected total object fluorescence (CTOF).

(1) TOF=Integrated density−(area of selected object ×mean flourescence of background readings) .

### 4.5. Cell Culture and MTT Assay

The human vascular endothelial cell line EA.hy926 was cultured in DMEM containing 10% FBS and 1% penicillin–streptomycin mixture. The cells were maintained in an atmosphere of 5% CO_2_ at 37 °C, and plates were split 1:3 when they reached confluence.

The cytotoxicity of IPA in EA.hy926 was assayed using the MTT assay. Briefly, 1 × 10^5^ of EA.hy926 cells were seeded in each well of 96-well plates. After incubation for 24 h, the cells were treated with different concentrations of IPA (0, 0.05, 0.15, 0.5, 1.5, and 2.5 µM), with three replicates for each concentration. After 24 h, the medium was replaced with 50 µL of MTT stock solution (2 mg/mL in PBS), followed by incubation for 3 h at 37 °C. The insoluble formazan product was dissolved in 100 µL of DMSO, and the absorbance was measured at 540 nm using a microplate reader (Synergy HT, BioTek Instruments, Winooski, VT, USA). Cell viability is expressed as a percent of the blank (no IPA treatment).

The effect of IPA on high glucose-induced cell proliferation was determined by measuring cell viability using the MTT assay. The cells were treated with 30 mM of glucose to induce angiogenesis [11]. The cells were treated simultaneously with glucose and different concentrations of IPA (0, 0.05, 0.15, 0.5, and 1.5 µM). After 24 h of treatment, cell viability was assessed using the MTT assay. Cell viability was expressed as a percent of the blank (0 mM glucose + 0 µM IPA). The effect of 30 mM glucose for cell viability was compared with that of the blank, and the effect of IPA on high glucose-induced cells was compared with that of the control (30 mM glucose + 0 µM IPA). 

In further experiments, the blank and control treatments were defined as follows: blank: 0 mM glucose + 0 µM IPA and control: 30 mM glucose + 0 µM IPA.

### 4.6. Scratch-Wound Cell Migration Assay

Cell migration was evaluated according to a previously described method with slight modifications [30]. EA.hy926 cells were seeded in 96-well plates, and the cells were grown to 80% confluence. The cell monolayer was scraped at the middle of the well using a sterile 10-µL pipette tip, and the cells were washed twice with PBS. The cells were treated together with glucose and IPA (0.15, 0.5, and 1.5 µM). After sample treatment, the cells were photographed (LIONHEART^FX^ automated live cell imager), and the initial gap length (0 h) was measured (Gen5 3.04). After 12 h of incubation, the final gap length was measured. The gap width was measured at five different places, and then it was averaged. To determine the effect for cell migration, the gap closure percentage was calculated as follows [11]:

(2) $$Gap\;closure\;\%=\frac{Initial\;gap\;length-final\;gap\;length}{initial\;gap\;lenth\;}\times 100$$

### 4.7. Transwell Migration Assay

Cell migration was evaluated using 8-µM pore-sized transwell filter chambers. Briefly, 100 µL of EA.hy926 cells was added to the upper chamber at a density of 3 × 10^4^ cells per well. The cells were subjected to different treatments with glucose and IPA (0.15, 0.5, and 1.5 µM) in serum-free media. Then, 500 µL of medium with 20% FBS was added to the lower chamber followed by incubation at 37 °C for 24 h. The cells on the upper side of the filter membrane were removed using cotton swabs. The cells on the lower side of the membrane were fixed by soaking in 4% paraformaldehyde for 30 min and stained with hematoxylin. Cell migration was determined by counting the stained cells at five different microscopic fields, and migrated cell percentage was calculated.

### 4.8. Determination of Matrix Metalloproteinase (MMP) Expression Levels Using the Enzyme-Linked Immunosorbent Assay (ELISA)

EA.hy926 cells were seeded in six-well plates at a density of 1 × 10^5^ cells/well. The cells were treated together with glucose and IPA (0.15, 0.5, and 1.5 µM). The culture media were collected after 48 h of incubation, and MMP (MMP-2 and -9) expression levels were evaluated using commercial ELISA kits, according to the manufacturer’s instructions.

### 4.9. Tube Formation Assay

EA.hy926 cells were seeded on top of Matrigel^®^ matrix to determine the effect of IPA in high glucose-induced capillary formation according to a previously described method [37]. Briefly, 96-well plates were coated with 75 µL of Matrigel^®^ per well and polymerized at 37 °C for 30 min. The trypsinized EA.hy926 cells were divided into approximately equal number of cells (1 × 10^5^), and the cell pellets were subjected to different treatments with glucose and IPA. After 6 h of incubation, cultures were photographed (4×) and analyzed using the plugin “Angiogenesis Analyzer” of image J software. Angiogenic score was calculated as follows [38]:

(3) Angiogenic score=Number of branches ×total branch length 

### 4.10. Western Blot Analysis

Protein extraction was performed separately for cytosolic proteins and membrane proteins, using a protein extraction kit (MEM-PER™ Plus Kit; Thermo Scientific, Waltham, MA, USA). The extracted proteins were quantified (Pierce™ BCA Protein Assay Kit; Thermo Scientific, Waltham, MA, USA), and equal amount of proteins (30 µg) were separated using 7.5% or 12% SDS-PAGE. The resolved proteins were transferred onto nitrocellulose membranes (GE Healthcare Life Science, USA) and blocked for 3 h with nonfat dry milk at room temperature. The membranes were then incubated overnight at 4 °C with the following primary antibodies: phosphorylated and/or total VEGFR-2, extracellular signal-regulated kinase (ERK), protein kinase B (AKT), c-Jun N-terminal kinase (JNK), endothelial nitric oxide synthase (eNOS), and glyceraldehyde 3-phosphate dehydrogenase (GAPDH). Following incubation with the secondary antibodies for 2 h, protein bands were detected using a chemiluminescence reagent (Maximum sensitivity substrate; Thermo Scientific, Waltham, MA, USA), and images were captured using Fusion Solo apparatus (Vilber Lourmat, Collégien, France). The relative levels of protein expression were measured using image J software and normalized to expression of the respective total form or GAPDH.

### 4.11. Statistical Analysis

The data were analyzed using GraphPad Prism 5 or Microsoft Excel and evaluated using two-way ANOVA and Dunnett’s multiple range tests. All the experiments were performed at least three times and expressed as mean ± standard deviation (SD). ns; not significant, * *p* ˂ 0.05, ** *p* ˂ 0.01, *** *p* ˂ 0.001, ^#^ *p* ˂ 0.05, ^##^ *p* ˂ 0.01, ^###^ *p* ˂ 0.001.

## 5. Conclusions

The findings of the present study demonstrated the anti-angiogenic effects of IO from a marine source. IO extract attenuated high glucose-induced vascular growth in transgenic zebrafish (flk:EGFP). IPA isolated from IO extract exerted anti-angiogenic effects against high glucose-induced angiogenesis. Furthermore, the anti-angiogenic mechanism of IPA in vascular endothelial cells showed that IPA suppressed high glucose-induced cell proliferation, cell migration, and capillary formation, which are known to be key steps involved in angiogenesis. IPA treatment suppressed VEGFR-2 receptor expression and downstream signaling cascade in high glucose-induced vascular endothelial cells. Thus, IO extract and IPA could be developed as potential therapeutic candidates for diabetes-related angiogenesis.

## Supplementary Materials

Supplementary materials can be found at https://www.mdpi.com/1422-0067/20/22/5542/s1. Figure S1: (A) HPLC chromatograms of IO extract and (B) IPA. The first 6 min in chromatogram is void time, Figure S2: Structure of Ishophloroglucin A (IPA) isolated from *Ishige okamurae*.
