# Supplementary material for: Ishige okamurae Extract and Its Constituent Ishophloroglucin A Attenuated In Vitro and In Vivo High Glucose-Induced Angiogenesis
Source: Int J Mol Sci. 2019 Nov 6;20(22):5542. doi: 10.3390/ijms20225542 (PMC6888214; doi:10.3390/ijms20225542)
Supplement: Supplementary file 1 [file ijms-20-05542-s001.pdf]

Supplementary

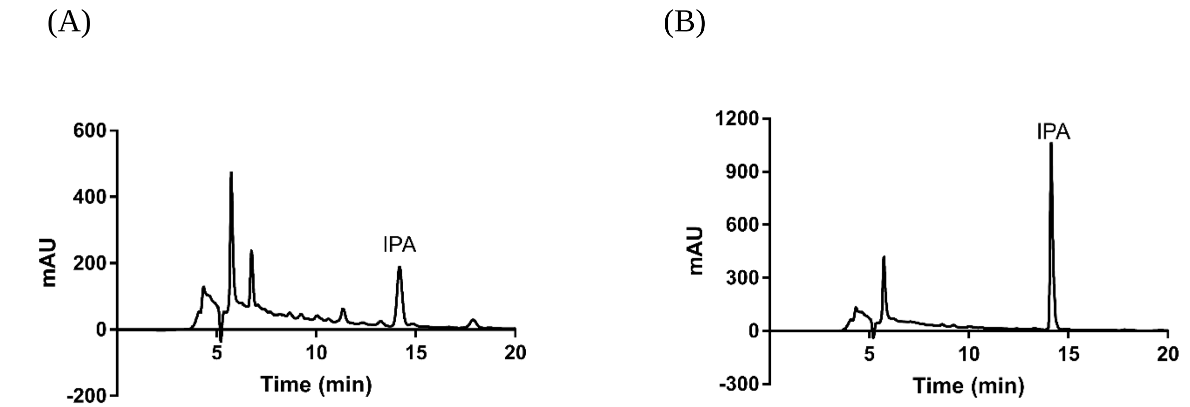

Supplementary Figure S1. (A) HPLC chromatograms of IO extract and (B) IPA. The first 6 mins in chromatogram is void time

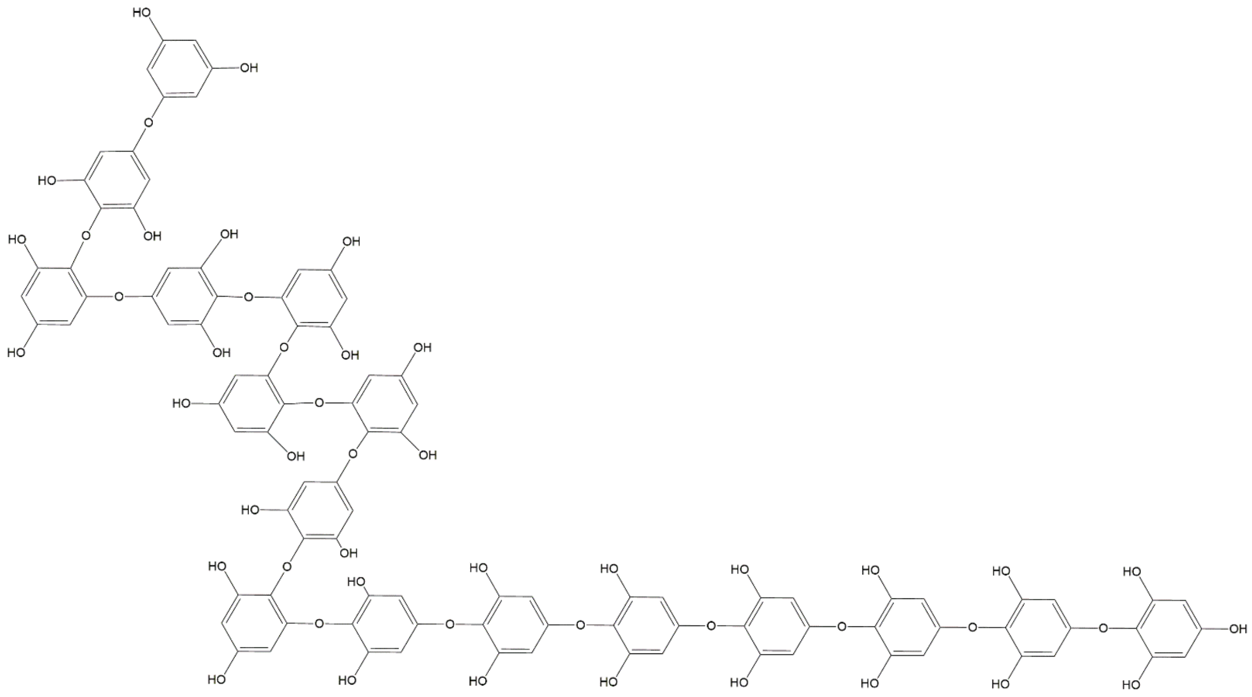

Supplementary Figure S2. Structure of Ishophloroglucin A (IPA) isolated from *Ishige okamurae.*

Supplementary materials and methods

High-performance liquid chromatography

The chromatographic analyses were conducted on an Agilent 1260 Infinity II gradient LC system VL (Agilent Technologies, Palo Alta, CA, USA) equipped with an Agilent Poroshell 120 EC-C18 column (4.6 mm × 150 mm, 4 μm) and a UV detector (230 nm). The mobile phase consisted of (A) 0.1% formic acid in water and (B) acetonitrile containing 0.1% formic acid. The HPLC eluting conditions was 31% of (B) for 30 min at a flow rate of 0.3 mL/min.
